# Electrochemical Formation and Characterization of Functional Ag-Re Coatings

**DOI:** 10.3390/ma18091893

**Published:** 2025-04-22

**Authors:** Oksana Bersirova, Valeriy Kublanovsky, Svetlana Kochetova, Olena Bondar

**Affiliations:** 1 Faculty of Chemistry and Geosciences, Vilnius University, 03225 Vilnius, Lithuania; 2 V.I. Vernadsky Institute of General and Inorganic Chemistry NAS of Ukraine, 03142 Kyiv, Ukraine

**Keywords:** silver, rhenium, electrodeposition, monoethanolamine, triethanolamine, functional coating, contact material

## Abstract

Silver-white, matte, smooth, and durable deposits of silver-rhenium, with thicknesses ranging from 2.0 to 13.7 μm and containing 0.15 to 13.5 wt.% Re, were obtained with a current efficiency of 66–98% from a developed dicyanoargentate–perrhenate bath based on a borate–phosphate–carbonate silver-plating electrolyte. This study was focused on the influence of bath composition, the [Ag(I)]:[ReO_4_^−^] ratio, surfactant additives, applied current density, temperature, and stirring, on the alloys’ composition, structure, morphology, microhardness, adhesion, and porosity. A voltammetric analysis was conducted, considering the influence of ethanolamines on electrode processes. In baths with triethanolamine (TEA), coatings similar to a silver matrix with rhenium doped in mass fractions are likely achievable. Monoethanolamine (MEA) is recommended due to its process-activating properties. All coatings were nanocrystalline (τ = 28.5–35 nm). For deposits containing less than 10 wt.% Re, characteristic silver XRD peaks were observed, while for other deposits, additional peaks attributed probably to Re(VII) and Re(VI) oxides. A linear relationship H_v_ − τ^−1/2^, typical for Hall–Petch plots, was obtained, confirming that grain boundaries play a crucial role in mechanical properties of coatings. The conditions for stable electrochemical synthesis of promising functional Ag-Re coatings of predetermined composition (0.7–1.5 wt.% Re) were proposed for practical use in power electronics and energy sectors for manufacturing electrical contacts operating across a wide temperature range. This was realized by deposition from an Ag-rich bath in the area of mixed electrochemical kinetics, at potential values corresponding to the region of half the limiting current: j = 2.5–6 mA cm^−2^, t = 19–33 °C.

## 1. Introduction

Silver-based composites and electrolytic alloys are prominent among thin-layer materials [1,2,3]. Electrolytic alloys of silver with rhenium [4,5], tungsten [6,7], and molybdenum are of interest both practically (e.g., as high-powered electrical contacts) and scientifically (because co-deposition of these metals allows the incorporation of refractory metals into alloys, which are difficult or impossible to obtain in pure form from aqueous solutions).

The interest in electrolytic alloys of silver with rhenium arises because of the combination of the unique electrical and catalytic properties of silver with the mechanical properties of rhenium [8,9]. The high melting point and exceptional resistance to recrystallization of rhenium have prompted its use in high-temperature environments like gas turbine superalloys, which represent its largest market [10,11]. Depositing Re alloys with metals like Ni, Co, Fe, In, Cr, Pd, Rh, Ag, or Au results in a significantly increased cathode efficiency (up to 80% or more) and a higher discharge potential for H^+^ ions [12,13,14].

According to [9], Kemp first proposed to use rhenium for electrical contacts due to its wear resistance in slip rings and switching contacts on motor commutators. Rhenium offers an alternative to traditional hard contact metals like Pt and Rh. Despite its industrial potential, rhenium has not gained widespread popularity in connector engineering. Its limited application in electric contacts is because of two factors: the high cost of rhenium and its tendency to form Re_2_O_7_ oxide, with a melting point of 569 K and a boiling point of 635 K [15]. This can lead to mass loss in contact materials due to erosion from the electric arc, though it also imparts high resistance to sticking [16]. However, it is indicated that is has sporadic but predominantly successful uses in specific applications, such as external engines of marine vessels, due to its resistance to seawater [15,17]. Recent research has focused on rhenium incorporating into contact materials to develop new generations of nanocrystalline materials like Ag-Re using high-energy grinding and careful plastic consolidation [16,18,19,20]. Findings related to the manufacturing technology and initial research on the physical, mechanical, and electrical properties of such materials was presented in [15].

For applications in power electronics and energy, specifically for manufacturing electrical contacts functioning across a broad temperature range (from −196 °C to +250 °C), functional silver-based alloys doped with rhenium, chromium, zirconium, and rare earth elements (cerium, lanthanum, yttrium) for applying wear-resistant and corrosion-resistant coatings using cold gas-dynamic spraying were developed [21]. Key to enhancing manufacturability and achieving the desired performance of the nanostructured silver-based alloy are, firstly, a judicious selection of the components of alloy, and, secondly, the creation of a nanocrystalline coating structure with superior corrosion and thermal properties. Moreover, the alloy must be lightly alloyed; to maintain the low electrical resistance of silver (the lowest among all metals 0.304 × 10^−8^ Ohm·m), alloying elements should not exceed 12 wt.%. The authors determined that cold resistance at −196 °C is achieved by adding 0.7 to 1.5 wt.% rhenium to the alloy. Increasing rhenium content above 1.5 wt.% reduced temperature stability (from +250 to +160 °C), while decreasing it below 0.7 wt.% diminished cold resistance at lower temperatures (down to −196 °C), leading to loss of mechanical strength in the coating (delamination, cracking). The electrochemical deposition method, with its ability to control factors like solution composition, current density, hydrogen ion concentration, and temperature, offers flexible and efficient control over the deposition process. However, despite rhenium-containing electrolytic alloys being highly promising, only a few studies on the electrodeposition of silver-rhenium coatings were carried out [22,23,24,25]. It was shown that multicomponent electrolytic coatings possess improved solderability, strength, and electrocatalytic activity, often displaying synergistic effects due to the properties of the constituent components. The developed coatings can be used in the electrical industry to produce contact groups for various purposes, including solid-lubricating wear-resistant coatings for friction pairs in vacuum, extreme temperature, and pressure conditions, and coatings with enhanced surface conductivity and reduced contact resistance.

Silver coatings, known for their low cost, high electrical conductivity, and corrosion resistance, are applied to both low-voltage and high-voltage contacts. High-voltage contact coatings must withstand electrical erosion and the elevated temperatures generated during contact opening and closing. Applying a finishing layer of silver-rhenium electrolytic coating [26] can improve the precision, reliability, wear, and corrosion resistance of contacting surfaces.

In the context of wear-resistant coatings for friction pairs, the electrochemical method for applying metal solid film lubricants is preferable due to several advantages over traditional processes using metal matrices. These include improved accessibility in physical areas, better control ensuring homogeneity and uniformity of coating, and enhanced adhesion reliability under various mechanical factors. Turns et al. [27] investigated a Ag-Re alloy plating process from a cyanide bath and electrodeposited Ag-3% Re alloy. The obtained 97% Ag-3% Re coatings may serve as solid lubricants withstanding high temperatures and loads. In addition, the highly porous plating serves dual functions: holding a high amount of MoS_2_ as grease and, once compacted under load, continuing to provide effective lubrication. Practical applications include aircraft wing hinge pins and the elimination of fretting corrosion and long-life contacts for rotary switches. Other baths for antifriction coatings consist of K_4_(Fe(CN)_6_), NaReO_4_, K_2_CO_3_, and monoethanolamine; a dissipative power of 53–57%, CE of 43–57%, and a rhenium content of 2–3% were determined [25].

To increase the rhenium content in the coatings [24], a self-regulating electrolyte was proposed having the following composition (in g L^−1^): KAgCN_2_—20–30; NaReO_4_—1–3; NH_4_NO_3_—15–20; and triethanolamine—2–3, pH 9.5–10.0, at room temperature. The cathode current density was set at 0.5–10 mA cm^−2^ without stirring and 10–40 mA cm^−2^ with stirring. The dissipative power of the electrolyte was 60–63%, and the current efficiency (CE) was 65–97%. A maximum of 20% rhenium in the coating was achieved from the stirred solution and at a high cathode current density of 40 mA cm^−2^. Under these conditions, the CE reached 85%, enabling the growth thickness of a coating up to 55 µm. So, developing new bath compositions for electrochemical co-deposition of silver-rhenium based on complex dicyanoargentate solutions is considered as most preferential.

As indicated in [6,7], the electrodeposition of thin-layer composite silver coatings via co-depositing of oxides of refractory metals is highly promising. The electrochemical reduction of ReO_4_^−^ was studied at pH ranging from 0 to 7 [28]. The reduction of ReO_4_^−^ to metallic Re involves multiple stages of sequential reduction of the intermediate products. It was found that low pH values and perrhenate concentrations favor the formation of the intermediate Re^3+^, which aids in forming metallic Re. Thus, the pathway of reduction is as follows: ReO_4_^−^ → ReO_3_ → Re^3+^ → Re.

Either at higher pH or ReO_4_^−^ concentration, it tends to form on the cathode the intermediate ReO_2_, which promotes the hydrogen evolution reaction and significantly impedes the reduction of low-valent intermediates. The corresponding reduction process is ReO_4_^−^ → ReO_3_ → ReO_2_, Re_2_O_3_. X-ray studies have indicated that NH_4_ReO_4_ plays a crucial role in the reduction process, but high concentration of NH_4_ReO_4_ is detrimental to obtain Re in metallic form because rhenium oxides ReO_3_ and ReO_2_ are formed [28].

It was determined [26] that co-deposited rhenium with silver in the film exits in form of ReO_x_. Deposition was conducted from a dicyanoargentate solution with the composition, g L^−1^: KAgCN_2_—20; NH_4_ReO_4_—1–5; NH_4_NO_3_—15; triethanolamine—5. The authors [26] achieved a maximum rhenium content in deposits of 0.7 wt.% using non-stationary reverse current, stirring, and ultrasound. These coatings exhibit high microhardness, wear, and corrosion resistance.

Controlled electrochemical formation of functional coatings from metals and alloys, including silver and its alloys with rhenium, having specific structures, can be achieved based on the understanding of the relationship between the electrochemical kinetics of electrode processes and electrodeposition [29,30,31,32]. Recently, from these perspectives, both the process of electrochemical deposition of silver [33,34,35,36,37] and the process of co-deposition of rhenium in alloys from complex baths [38,39,40] have been studied. The main principles of forming coatings of binary and ternary rhenium-containing alloys for various functional purposes have been established. However, there are practically no studies in the literature dedicated to the process of electrochemical synthesis of functional Ag-Re coatings.

A non-toxic dicyanoargentate–borate–phosphate–carbonate (BPC) silver plating electrolyte has been developed [29]. BPC electrolyte does not contain “free” cyanide-ions, and its stability is ensured by a borate–phosphate–carbonate buffer. The mechanism of silver recovery from BPC electrolytes has been studied [29]. The electrolyte allows for high-quality coating production with strong adherence to the substrate even after prolonged bath operation. It has been effectively incorporated into the technology for functional silver plating of micro- and nanoelectronics components. Therefore, for the electrochemical deposition of silver coatings with rhenium, we consider the previously developed complex buffer BPC electrolyte as the most promising for developing co-deposition bath compositions. The key ingredients of the new bath for Ag-Re alloy electrodeposition could be the functional silver plating BPC electrolyte and ammonium perrhenate as the rhenium source because it is commonly used for electrodeposition of rhenium and its alloy [41,42].

This study presents a novel and original approach to the formation of silver-rhenium coatings, based on the determination of a stable electrodeposition process range within the mixed electrochemical kinetics regime. This approach contributes to the advancement of the field of electrolytic deposition of metals and alloys with a controlled composition.

This work aimed to investigate the process of electrodeposition of silver-rhenium coatings from a dicyanoargentate–perrhenate bath, developed based on a borate–phosphate–carbonate buffer electrolyte for functional silver plating, and to characterize the structure and properties of obtained coatings.

## 2. Experimental Section

### 2.1. Bath Composition and Procedure

The study of electrodeposition of Ag-Re coatings was conducted using dicyanoagentate-perrhenate plating solutions. These solutions were prepared by adding ammonium perrhenate to borate–phosphate–carbonate (BPC) silver plating electrolyte [29], which contains KAg(CN)_2_, and buffers such as KH_2_PO_4_, K_2_HPO_4_, H_3_BO_3_, K_2_CO_3_, and KOH (pH 7.0 ± 0.1). The choice of ammonium perrhenate as the rhenium ion source is due to its higher solubility in the operational temperature range compared to KReO_4_ (3.25 × 10^−2^ vs. 5.08 × 10^−3^ g mol/100 g H_2_O at room temperature) [43]. The specific compositions of these electrolytes are detailed in Table 1.

Electrodeposition occurred from baths with a constant silver concentration (KAg(CN)_2_—20 g L^−1^) but varying concentrations of perrhenate ions (bath #1, 2, 5, 6, 7). The [Ag^+^]:[ReO_4_^−^] ratio was 10:1 (bath #2 and 4) and 2:1 (bath #5, 6, 7). Additionally, experimental studies were conducted in solutions where the silver concentration was reduced and [Ag^+^]:[ReO_4_^−^] ratio was 1:1.5 (bath #8); 1:5 (bath #3) and 1:10 (bath #9 and 10); as shown in Table 1.

The electrolysis conditions were also diversified, including a range of current densities (3, 5, 7.5, 10, 15 mA cm^−2^) and temperatures (18, 20, 30, 40, 50, 60 °C), both without and with stirring using a magnetic stirrer.

All electrolytes were prepared using double-distilled water and analytical grade reagents. The process began with dissolving a sample of potassium dicyanoargentate (KAg(CN)_2_) in double-distilled water. Then, a borate–phosphate–carbonate (BPC) buffer was added to this solution, enhancing the ionic strength and stabilizing ion states in both the electrolyte volume and cathode layers during electrolysis. The pH of the BPC electrolyte was maintained at 7.0 ± 0.1 and adjusted using boric acid. The required amount of ammonium perrhenate was added to the dicyanoargentate solution containing the BPC buffer, with careful pH monitoring. In experiments studying the effect of surfactants, specific concentrations (0.11 M KAg(CN)_2_ and 0.055 M KReO_4_) were used, with incremental additions of monoethanolamine (MEA) and triethanolamine (TEA) ranging from 0.1 to 0.5 mL (Table 1). For baths with ethanolamine additives, ammonium perrhenate was replaced with potassium perrhenate. KReO_4_ was chosen to avoid additional cations and to use silver and rhenium salts with the same cation. The literature suggests that ammonium rhenium salt can slightly increase deposit porosity compared to potassium or sodium salts [44].

### 2.2. Electrochemical Measurements

Experiments were conducted in a 50 mL standard electrochemical glass cell within a temperature range of 20 to 60 °C, using a UTU-4 thermostat (ZEAMiL HORIZONT, Cracow, Poland) with an accuracy of ±0.1 °C. An electric magnetic stirrer at 120 rpm was used for mixing the electrolyte.

For voltammetric studies, a platinum wire, 1 mm in diameter and sealed in glass, served as the working electrode. Both platinum and electrochemical silver were used as working electrode materials. To prepare the silver electrode, the platinum wire’s end was coated with a 1.5-micron thick electrolytic silver layer at a polarization of 0.150 V. All potential measurements were relative to a Ag/AgCl reference electrode in a saturated potassium chloride solution, with all potentials reported relative to this silver chloride reference electrode.

Voltammograms were recorded using a PC Controlled Potentiostat, type EF453 “Electroflex” (ELECTROFLEX, Budapest, Hungary), at temperatures of 20, 25, 30, 35, and 40 °C. Potentiodynamic polarization dependencies were captured over a broad range of scan rates from 1 to 100 mV s^−1^. Each curve was recorded multiple times, ensuring good reproducibility of the results. When comparing curves from parallel experiments conducted under identical conditions, a complete alignment was observed. Prior to experimentation, the electrode was immersed in the solution for 30 min to establish an equilibrium potential. The initial sections of the polarization curves, obtained at a scan rate of 1 mV s^−1^, were converted into semilogarithmic coordinates for determining the kinetic parameters of the electrode process. The activation energy was calculated from the slope of the inverse temperature dependence of the exchange currents.

The electrodeposition was performed in galvanostatic mode; the electrolysis times were set to either 30 or 60 min. A copper plate with a 2 cm^2^ area served as the working electrode for coating electrodeposition. Silver wire or glassy carbon were used as anodes.

Current efficiency was calculated based on the mass of the obtained deposit, the passed charge, and the chemical composition of the deposit determined by the EDX method. The calculation utilized the following equation:

(1) $$CE=\frac{\Delta m}{It}\Sigma \frac{wt{\%}_{i}\cdot n_{i}\cdot F}{M_{i}}\cdot 100\%,$$

where ∆*m* is the measured weight of the deposit (g), *t* is the deposition time (s), *I* is the total current passed (A), *wt*%*_i_* is the weight fraction of the element (Ag or Re) in the deposit, *n_i_* is the number of electrons transferred per atom each metal (*n_i_* = 1 and 7 for Ag and Re, respectively), *M_i_* is the atomic mass of that element (*M_i_* = 107.6 and 186.2 g mol^−1^ for Ag and Re, respectively), and *F* is Faraday’s constant (96,485 C mol^−1^).

The coating thickness *δ* was calculated based on the weight gain of the sample after application, the chemical composition of the deposit (wt.%), and the density of each metal according to the formula:

(2) $$\delta =\frac{∆m}{\rho *S}=\frac{\;∆m}{\sum \left(\rho_{i}*wt{\%}_{i}\right)*S}\;,$$

where *ρ_i_* is the density of the element (Ag and Re) (kg dm^−2^); *wt%_i_*—mass fraction of silver and rhenium, respectively; ∆*m*—is the measured weight of the alloy mass (g); *S*—cathode area (cm^2^).

### 2.3. Surface Morphology, Chemical Composition and Crystallographic Structure Characterization

The morphology and chemical composition of the Ag-Re deposits were analyzed using SEM and EDX methods. This involved a scanning electron microscope (JSM 6700F, JEOL Ltd., Tokyo, Japan) equipped with an energy dispersive spectrometer (JED-2300), operating at an accelerating voltage of 20 kV, a beam current of 0.75 nA, and a beam size of 1 µm. For EDX analyses, the calculation time was set to 1 min.

The structure of the electrodeposited silver and Ag-Re alloys was investigated by X-ray diffraction (XRD) methods (Rigaku MiniFlex II, Tokyo, Japan). XRD patterns were produced with Cu Kα radiation (1.5406 Å) in 2θ scanning mode from 20 to 90° with a step of 0.01°. For most precise θ values for peaks and line broadening determination, the routines of the peak fitting using Gaussian- or Lorenzian-type functions were performed.

The crystallite sizes were calculated using the Scherrer formula:

τ = K λ/βτ cosθ(3)

where βτ is the peak broadening (in rad); K is the Scherrer factor—the empirical constant 0.9; τ is the crystalline size (in Å).

### 2.4. Microhardness, Adhesion and Porosity Measurements

The Vickers microhardness of the samples was measured with a microhardness tester PMT-3 (Vostok-7, Moscow, Russia) under a load of 10 g and a load exposure of 10 s. Arithmetic mean values of H_V_ show the trend between the samples.

The adhesion of the coatings was tested using the “scotch tape test” according to ISO 2409 standard [45] methodology.

The porosity of the coatings was assessed by applying filter paper moistened with a solution containing (g L^−1^): K_3_[Fe(CN)_6_]—3, NaCl—10.

Each test was carried out multiple times, ensuring good reproducibility of the results.

## 3. Results and Discussion

### 3.1. Voltammetric Studies

The study of deposition kinetics makes it possible to establish the patterns of chemical and electrochemical reactions depending on time, current density, concentration of additives, and other factors affecting the rate of both the overall process and intermediate stages [6,46]. Such studies are necessary to consider the patterns of changes in the rate of electrode processes, which are influenced by the passage of electric current through the “electrode-ionic system” boundary, charging of the electrical double layer, and the distribution of ions in the volume of the electrolyte.

#### 3.1.1. Results of Studies of Stationary Potential

The measured stationary potentials for silver electrode in different baths for the deposition of Ag-Re coatings are shown in Table 2.

The introduction of ammonium perrhenate into the BPC silvering electrolyte based on potassium dicyanoargentate affects the stationary potential of the silver electrode, changing it to the cathodic side by 222 mV to −0.022V (vs. Ag/AgCl) in the case of adding 0.011 M NH_4_ReO_4_, (bath #2) and by 193 mV to +0.007 (vs. Ag/AgCl) when adding five times more perrhenate (bath #5). The influence of the concentration of dicyanoargentate ions can also be traced. Thus, decreasing the concentration from 0.11 M (bath #2) to 0.022 M KAg(CN)_2_ (bath #4) shifts the potential to the cathodic side by 176 mV, and to 0.004 M KAg(CN)_2_—by 322 mV to −0.122 V (vs. Ag/AgCl) (bath #3).

The addition of 0.06 M triethanolamine (bath #7) has little effect on the stationary electrode potential, which remains the same as in a solution of the same concentration of silver and rhenium ions, but without surfactant additives (bath #5). Unlike triethanolamine, monoethanolamine depolarizes the electrode from +0.007 V (in bath #5) to +0.170 V, although the concentrations of both silver and rhenium in solution remain unchanged. In the dicyanoargentate–perrhenate solution, the stationary potential of the silver electrode is the same with the stationary potential in the BPC silver plating electrolyte of the same concentration of silver ions.

#### 3.1.2. Results of Studies of Potentiodynamic Polarization Curves

**Figure 1:**
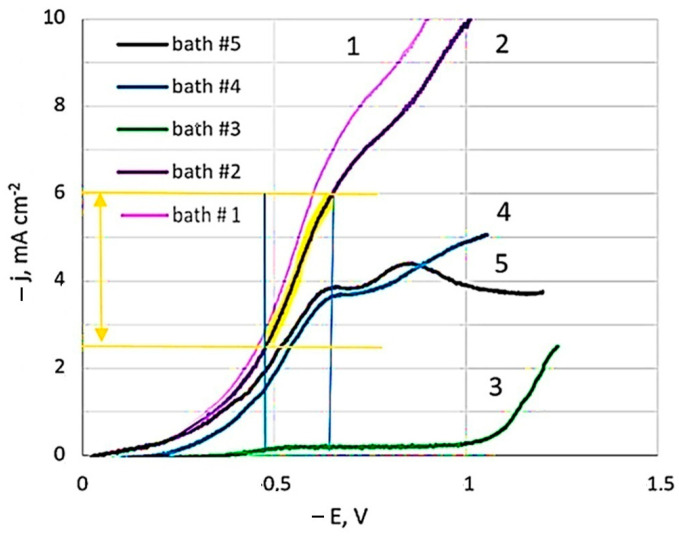
Cathode polarization curves depending on the concentration of baths components. The temperature is 20 °C. Potential sweep rate of 1 mV s^−1^.

Linear sweep voltammetry studies were carried out in the developed dicyanoargentate–perrhenate plating solutions showed in Table 1. Figure 1 presents polarization curves for pure silver (bath #1) and those obtained from baths with different [Ag^+^]:[ReO_4_^−^] component ratios: 10:1 (bath #2, #4); 2:1 (bath #5); and 1:5 (bath #3).

In the study of polarization curves at a potential sweep rate of 1 mV s^−1^ in a dicyanoargentate–perrhenate electrolyte, a limiting current plateau of 6.8 mA cm^−2^ (bath #2) was observed. The polarization curve in the Ag-rich bath, with rhenium concentration ten times less than silver (bath #2), closely resembled that of the BPC silver bath without perrhenate (bath #1), albeit slightly lower. Limiting current potentials were consistent across all curves. However, adding rhenium, even at a lower concentration compared to silver, slightly reduced the limiting current values. Comparing baths with equal silver concentration but five times higher rhenium concentration (curve 5 vs. curve 2) revealed even lower limiting current values. With the same silver to rhenium ratio (10:1) but diluted fivefold (curve 4 vs. curve 2), a significant decrease in limiting current values was noted. Bath #3, having 25 times less silver than the initial bath #2, showed the minimal limiting currents among all studied solutions. Curves 4 and 5 distinctly displayed two sections of limiting currents, while the second limiting current was not as pronounced in the other curves.

Voltammograms in semilogarithmic coordinates were used to calculate kinetic characteristics (apparent transfer coefficients, exchange current densities) at different temperatures. Adding NH_4_ReO_4_ salt to the BPC electrolyte reduced the exchange current value, leading to lower effective current density. The apparent transfer coefficient in bath #2 was 0.170 ± 0.005, indicating minimal influence of the electrode electric field (double layer energy) on activation energy [47]. Formal exchange currents increased nearly tenfold with temperature rising from 20 to 40 °C. The activation energy, calculated from the exchange currents against reverse temperature, was 65 kJ mol^−1^ in bath #2, closely matching the value of 65.6 kJ mol^−1^ determined for electroreduction of silver from a BPC electrolyte without ammonium perrhenate (bath #1).

**Figure 2:**
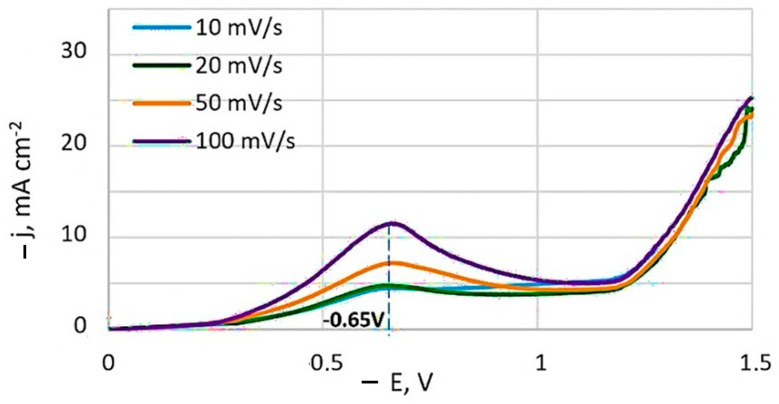
Potentiodynamic cathodic curves obtained at a temperature of 20 °C from bath #4. Potentials are given in relation to the Ag/AgCl reference electrode.

Typical potentiodynamic voltammograms obtained at different potential sweep rates is depicted in Figure 2. A current peak is present on the non-stationary curves, and its value increases with the sweep speed. The potential of this current peak remains essentially constant at −0.65 V, relative to the Ag/AgCl reference electrode.

**Figure 3:**
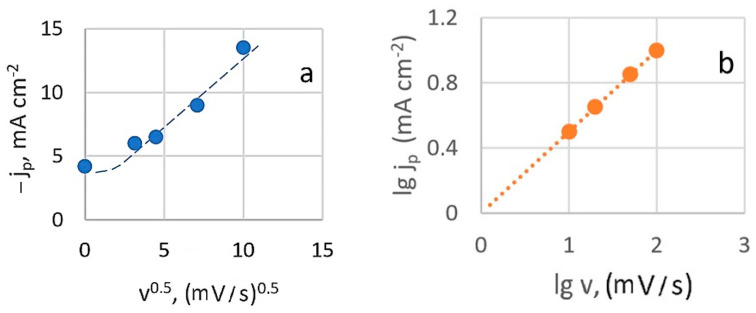
Dependence of the peak current on the scan speed in criterion coordinates: peak current is the square root of the scan speed (**a**) and logarithmic dependence (**b**).

At slower charge transfer stages, the concentration of depolarizer on the electrode surface is governed by the rate constant. When a potential sweep is applied, the sweep rate also influences this concentration. Figure 3a shows that the relationship between the height of the peak cathode current and the square root of the potential sweep rate (at 20 °C) is generally linear. However, deviations from linearity at low sweep speeds are observed, which can be attributed to the effects of natural convection.

This suggests that the diffusion process is irreversible and indicates that the electroactive complex is likely restored from an adsorbed state under these conditions. However, when the current peak dependence on sweep rate is plotted in logarithmic coordinates (Figure 3b), the calculated slope value (lg*j_p_*/lg*v*)*_t,C_* is 0.5. This rate dependence typically indicates that processes are limited by charge transfer rate, diffusion, and kinetics of a chemical reaction (mixed kinetics). It excludes catalytic processes and those involving adsorption complexities.

#### 3.1.3. The Effect of Surfactants on the Electrode Process

The impact of organic nitrate surfactant additives, specifically monoethanolamine (MEA) and triethanolamine (TEA), was examined. Although the use of these additives in baths for forming functional Ag-W (or tungsten oxides) and Ag-Re (or rhenium oxides) coatings are referenced [6,7,26], their influence on the electrode process has not been detailed.

In developing the optimal composition of BPC silver electrolytes, various additives like ethylenediamine, glycine, methylamine, ethylamine, dimethylamine, dicyclohexylamine, EDTA, KNO_3_, NH_4_NO_3_, K_4_P_2_O_7_ among others, were studied. It was found that ethylenediamine and nitrates are more promising for facilitating anodic processes [29]. However, the effects of MEA and TEA had not been explored, prompting this investigation.

**Figure 4:**
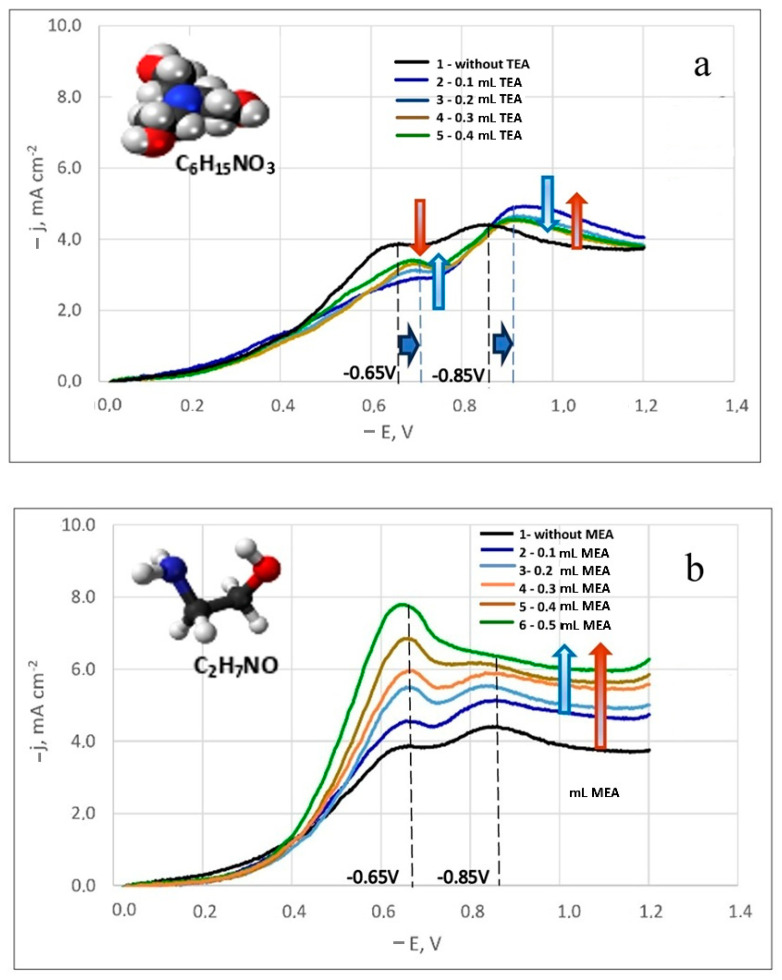
Potentiodynamic cathodic curves in dicyanoargentate–perrhenate silver electrolytes with TEA (**a**) and MEA (**b**) additives. The temperature is 50 °C. Potential sweep rate is 100 mV/s.

A noticeable difference in the influence of these surfactant additives on the electrode process was observed. Figure 4a,b displays the polarization curves for silver-rhenium co-deposition in solutions with surfactant additives. The numerals adjacent to the curves indicate increasing amounts of added surfactants. It was observed that an increase in MEA concentration led to higher maximum discharge currents of electroactive ions (Figure 4b), while TEA addition resulted in decreased currents (Figure 4a).

Although the equilibrium potential in these electrolytes remained unchanged, a shift in the current peak potentials was noted in the solutions containing TEA addition. For both the first and second peaks, the potential shifted by 50 mV towards more negative potentials (Figure 4a). Furthermore, the first limiting current values for all studied TEA concentrations were lower than those in a similar composition solution without additives (bath #5). With increasing TEA concentration, the first limiting currents increased slightly. For the second peak current, the values were higher than in bath #5, but they decreased slightly with increasing TEA concentration. This inhibition of the electrode process by TEA can be attributed to its potential adsorption on the surface of the coated substrate, obstructing the surface and hindering the reduction of electroactive dicyanoargentate ions due to possible competitive adsorption.

With the addition of MEA, there was a significant increase in the first peak currents, proportional to the MEA concentration. Initially, the second peak currents also increased slightly before the second limiting current peak leveled off (Figure 4b). For all concentrations studied, the curves were higher than the polarization curve obtained in bath #5. In an electrolyte solution, MEA can function as an additional ligand. Since the primary ligand, the [CN]^−^ group, is bound, MEA^+^ ligands help prevent the passivation of silver anodes by forming an intermediate silver(I) complex with monoethanolamine ([Ag(MEA)]^+^ pK_1_ = 3.1, [Ag(MEA)_2_]^+^ pK_2_ = 3.5 [48]), followed by the formation of a strong complex [Ag(CN)_2_]^−^ pK = 21.1 [29,35] in the solution. The electrode process activation by MEA is likely due to its complexing abilities with silver, potentially leading to the formation of a complex polyligand silver compound.

This behavior of surfactant additives in the developed dicyanoargentate–perrhenate electrolyte can be leveraged to create various types of coatings. It is recommended to use MEA as a surfactant in the dicyanoargentate–perrhenate electrolyte due to its process-activating properties. In baths with TEA, coatings akin to a silver matrix with rhenium doped in fractions of a mass percent are likely achievable. For further studies on silver-rhenium electrodeposition, the maximum concentrations of MEA and TEA were chosen. The following sections present results regarding the deposition composition, structure and morphology, and some properties.

### 3.2. Electrodeposition of Ag-Re Coatings

#### 3.2.1. Dependency of Deposit Composition and Morphology on Current Density and Hydrodynamic Electrolysis Condition

**Figure 5:**
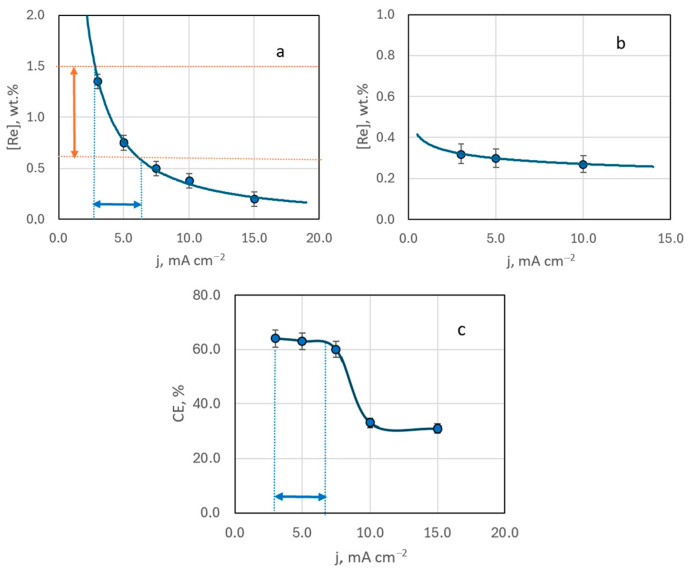
The mass fraction of rhenium in the deposit (**a**,**b**) and current efficiency (**c**) depending on the deposition current density. Electrolysis temperature, °C: 20—(**a**,**c**); 60—(**b**).

EDX analysis of samples obtained from Ag-rich bath #2 ([Ag^+^]:[ReO_4_^−^] ratio of 10:1) indicated that the resulting deposits contain up to several mass percent of rhenium. An increase in deposition current density from 3 to 15 mA cm^−2^ led to a decrease in rhenium content in the deposit (Figure 5a). This trend was consistent across samples deposited at different temperatures (20 to 60 °C), regardless of stirring (Figure 5b). The highest rhenium content in the deposit, 1.35 wt.%, was achieved with electrodeposition at a current density of 3 mA cm^−2^, at room temperature, without stirring.

It was observed that the oxygen content in most samples was approximately three times higher than the rhenium content, suggesting that rhenium in the coatings exists predominantly in the form of ReO_x_. This raises the possibility of forming composite “silver–rhenium oxides” coatings, although further research is required to confirm this.

Current efficiency also decreased with increasing deposition current density. Up to 10 mA cm^−2^, the efficiency remained relatively constant, but at higher currents, it dropped by almost half and then stabilized (Figure 5c).

According to our previous studies [29], the range for stable production of functional coatings with a specified composition should correspond to the conditions of mixed electrochemical kinetics and is achieved at potentials corresponding to half the limiting current area.

Analysis of the voltammetric polarization curve (bath #2) allowed us to identify the corresponding range of operating current densities for the studied system (Figure 1). At 20 °C, this range of deposition current densities was 2.5–6 mA cm^−2^. We highlight it in Figure 5a. It has been experimentally established that the deposits synthesized under these conditions contain strictly 0.7–1.5 wt.% Re. Such coatings are very interesting from a practical point of view. Since the electrodeposited silver alloys are lightly alloyed (up to 12 wt.% Re), the low resistance of the silver base is maintained. They can be promising for replacing electrical contacts obtained by the micrometallurgical method [21], which can operate in a wide temperature range (from −196 °C to +250 °C) without losing their properties. Hence, we conclude that these conditions for electrochemical synthesis are technologically advantageous and refer to them as the “functional electrodeposition range”.

**Figure 6:**
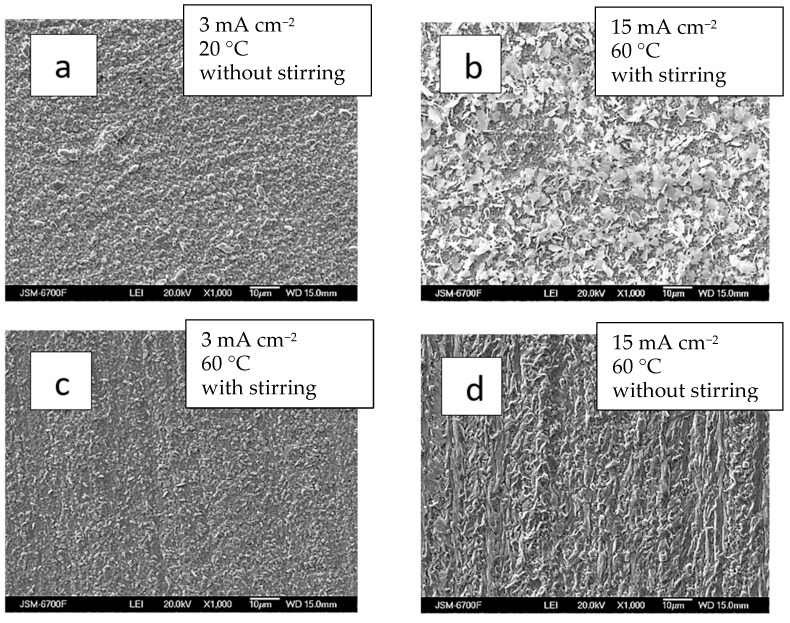
SEM images of samples obtained from bath #2 at different current densities, mA cm^−2^: 3—(**a**,**c**); 15—(**b**,**d**); and temperature, °C: 20—(**a**); 60—(**b**–**d**); without (**a**,**d**) and with stirring (**b**,**d**). The thickness of the coatings is 2–2.5 µm.

The coatings were consistently silver-white and fine-crystalline, without burns or spots, across the current density range of 3 to 15 mA cm^−2^. They were dense, evenly distributed, and uniformly structured. At lower current densities (3 mA cm^−2^) and room temperature, the Ag-Re deposit exhibited a uniform, closely packed, and finer crystalline structure (Figure 6a).

The thin-layer Ag-Re coatings are uniformly silvery white, fine-crystalline, and matte. The coatings are well adhered to the copper substrate. Upon SEM analyzing the surface morphology, it was observed that most deposits closely replicate the copper substrate.

Chemical analysis revealed that despite the thickness of the coatings, the electrodeposits cover the substrate almost entirely. The deposition current density plays a role comparable to stirring in determining surface morphology. For example, at temperature 60 °C, the morphology of coatings obtained at 15 mA cm^−2^ with stirring resembled those formed at the same current density in a bath without stirring (Figure 6b,d).

The surface morphology of Ag-Re coatings obtained at different current densities and under intensifying factors such as stirring and elevated temperature (up to 60 °C) varies slightly with different applied deposition current densities (ranging from 3 to 15 mA cm^−2^) (Figure 6b,c). Appearance of the coatings obtained at higher current density (15 mA cm^−2^) are somewhat distinct from others.

#### 3.2.2. Dependency of Deposit Composition and Morphology on Bath Temperature

**Figure 7:**
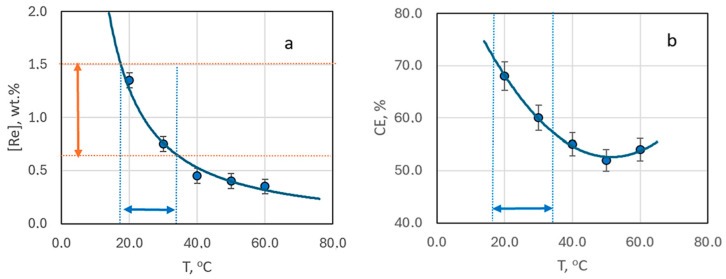
The mass fraction of rhenium in the deposit (**a**) and current efficiency (**b**) depending on the deposition temperature. The samples obtained from bath #2 at current density 3 mA cm^−2^ without stirring.

Comparing the rhenium content in deposits obtained at a constant current density in an electrolyte at a [Ag^+^]:[ReO_4_^−^] ratio of 10:1, without forced convection but at varying temperatures from 20 to 60 °C, reveals a trend of decreasing rhenium content with rising temperature (Figure 7a).

The reduction in rhenium content is most pronounced when the electrolysis temperature increases from 20 to 30 °C. In this case it drops almost threefold. Beyond this, the decrease in rhenium content becomes more gradual, with little change at temperatures between 40–60 °C (Figure 7a). Interestingly, the current efficiency demonstrates a dip, reaching a minimum at 50 °C, before increasing again (Figure 7b). SEM images of the coating surfaces obtained at 50 °C (Figure 6b) differ from those produced at other temperatures.

Figure 7a identifies the optimal temperature range (19–33 °C) for achieving coatings with a rhenium content in the “technologically advantageous” range of 0.7–1.5 wt.%.

#### 3.2.3. Dependency of Deposit Composition and Morphology on Electrolyte Component Ratios

**Figure 8:**
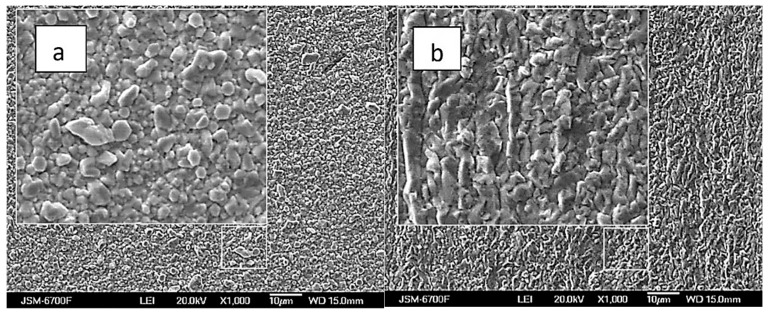
SEM images of samples obtained from baths with the [Ag^+^]:[ ReO_4_^−^] ratio: 10:1 (**a**), and 1:5 (**b**). Current density 10 mA cm^−2^, temperature 20 °C.

The silver and rhenium ion content ratio in the solution significantly influences the structure and morphology of Ag-Re deposits. Figure 8a,b illustrates that coatings from baths with a higher ratio of rhenium ions are more densely packed. In contrast to the white matte coating observed in baths with a predominance of silver, where scattered crystals of similar size are noticeable, the coatings from rhenium-rich baths appear more “metallic” and shiny. Nevertheless, both the deposit composition and the coating surface are more sensitive to factors like deposition current density and temperature.

**Figure 9:**
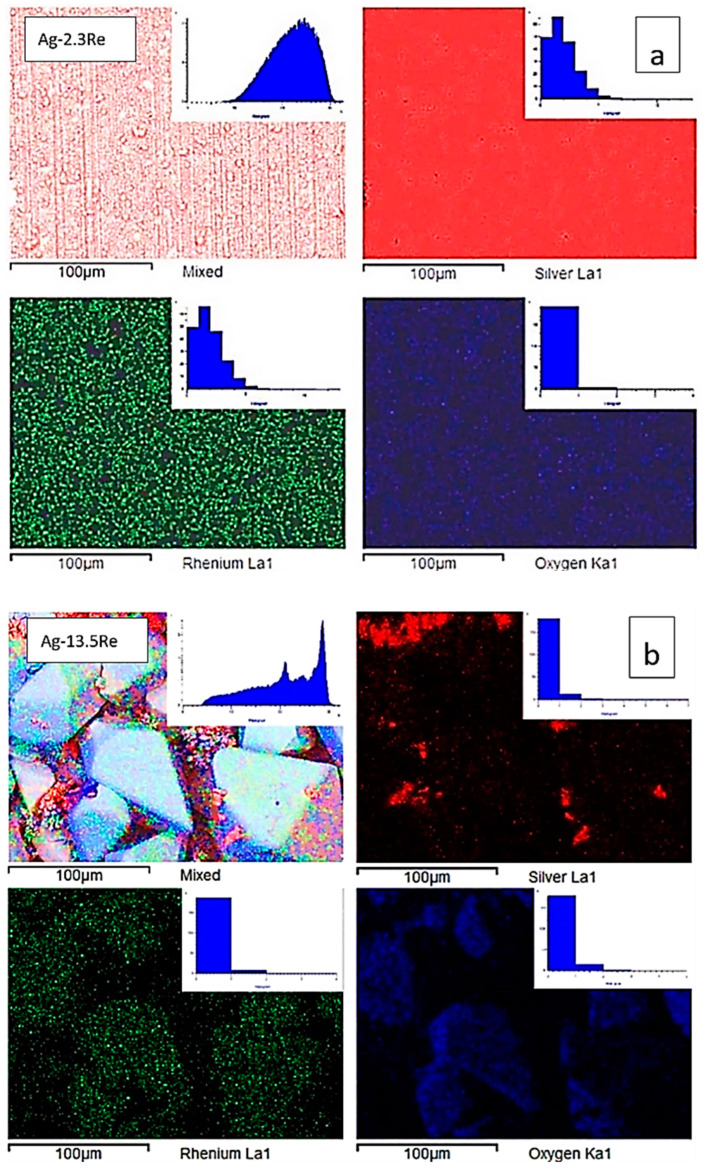
SEM images, corresponding elemental maps of Ag, Re, and O of sintered samples Ag-2.3Re (**a**) and Ag-13.5Re (**b**).

It should be noted that the morphology of deposits obtained from Re-rich baths differs from Ag-rich baths. Analysis of the SEM-EDS mapping performed (Figure 9) demonstrates a uniform distribution of elements for samples deposited from solution with ratio [Ag^+^]:[ReO_4_^−^] = 10:1 (bath #2), and uneven for coatings deposited from the highest Re-rich solution with ratio [Ag^+^]:[ReO_4_^−^] = 1:10 (bath #10). This is clearly seen from the histograms of the distribution of elements shown in the same figure.

Deposits obtained from the Re-rich bath (bath #10) contain areas where dendritic silver is deposited, as well as disordered large (up to 85 μm) macrocrystals, which, as can be seen from the distribution of elements, consist mainly of rhenium and an oxygen containing phase. This indicates that Ag-Re deposits containing more than 10 wt.% Re may be a composite of silver and rhenium oxides. According to EDX analysis in the point of macrocrystal, the ratio of the atomic fractions of rhenium and oxygen did not allow us to unambiguously assume the stoichiometric composition of the oxide phase.

#### 3.2.4. Dependency of Deposit Composition and Morphology on Surfactant Addition

Based on a voltammetric study, baths with higher ethanolamine additive content were chosen for further investigation. To evaluate the effect of ethanolamines additives on deposit composition and morphology, coatings were electrodeposited from baths with a silver to rhenium ion ratio of 2:1 (bath #5), with addition of 1.65 g L^−1^ monoethanolamine (bath #6) and 0.6 g L^−1^ triethanolamine (bath #7).

**Figure 10:**
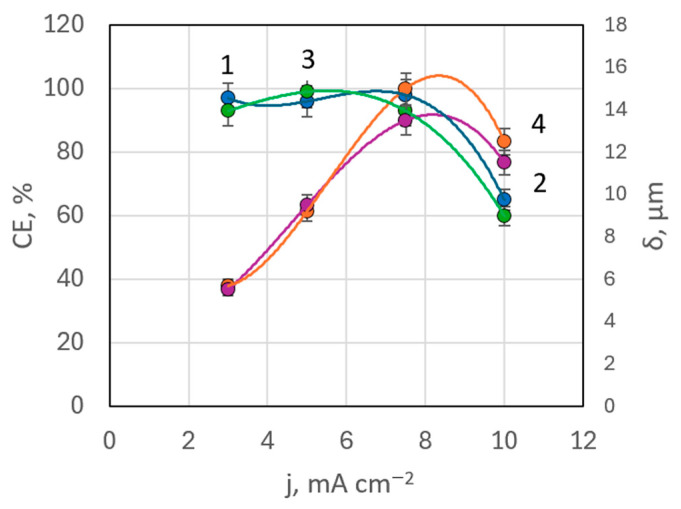
Dependences of current efficiency (curves 1, 3) and coating thickness (curves 2, 4) on deposition current density in the presence of TEA (curves 1, 2) and MEA (curves 3, 4).

EDX analysis indicated that in bath #6 (with MEA) at 50 °C, the maximum rhenium content in deposits was 0.568 wt.%, whereas in bath #7 (with TEA) at the same temperature, rhenium content ranged between 0.17–0.485 wt.%. These values were lower than in baths without surfactants. There was not a clear correlation between rhenium content in the coating and deposition current density in these baths. Current efficiency remained high, almost constant up to a current density of 7.5 mA cm^−2^, but then drops down to 66% at higher current densities (Figure 10). This pattern was observed with both MEA and TEA additives.

A slight decrease in rhenium content in the deposits from solutions with equal silver concentration but a fivefold higher concentration of perrhenate may be partially attributed to the increased pH levels in baths with ethanolamine additives. The pH was increased from 7.02 in bath #5 to 8.85 in bath #6 (with MEA) and 8.34 in bath #7 (with TEA), as shown in Table 1.

In baths with ethanolamine additives, it was possible to produce coatings with thicknesses ranging from 5.0 to 13.7 microns. Deposits formed at lower current densities were silvery-white and of high quality, but dendritic growth was observed at current densities above 10 mA cm^−2^.

**Figure 11:**
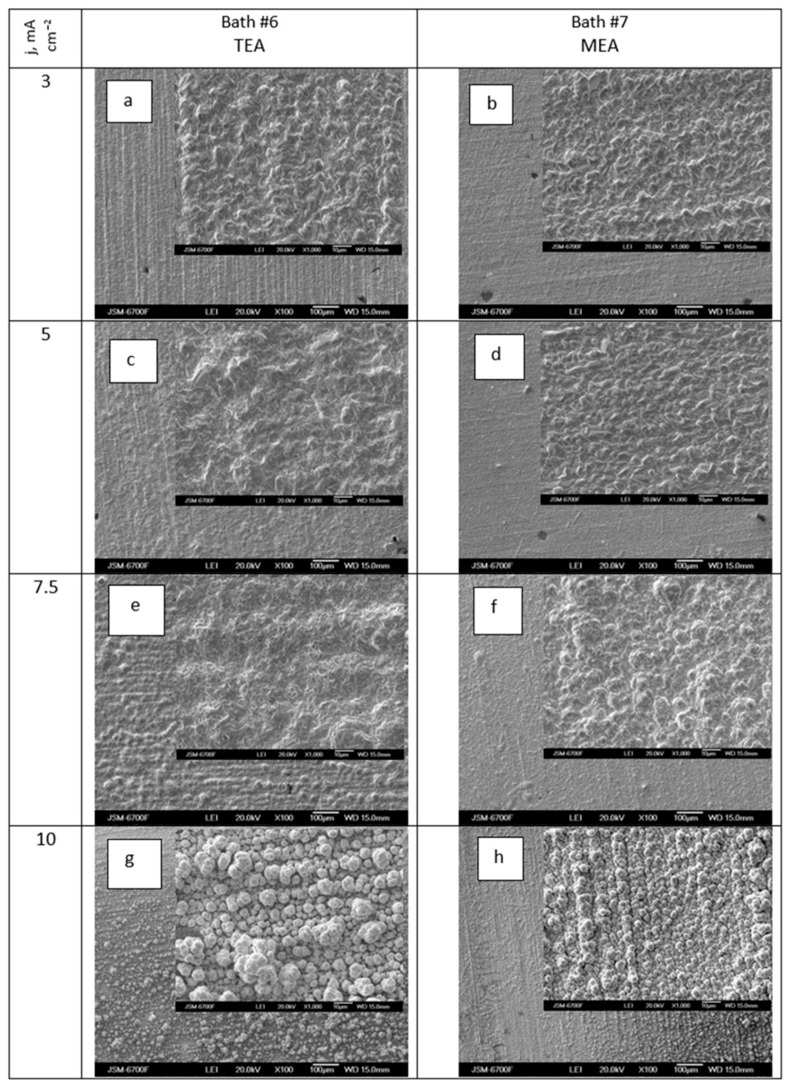
SEM images of samples obtained in dicyanoargentate–perrhenate electrolyte (KAg(CN)_2_–0.11 M; KReO_4_–0.055M) with additions of 0.06 mol L^−1^ triethanolamine (**a**,**c**,**e**,**g**) and 0.165 mol L^−1^ monoethanolamine (**b**,**d**,**f**,**h**) at current density, mA cm^−2^: 3 (**a**,**b**); 5 (**c**,**d**); 7.5 (**e**,**f**); 10 (**g**,**h**). Temperature 50 °C.

Figure 11a–h demonstrates the impact of deposition current density on the morphology of Ag-Re coatings obtained from a silver dicyanoargentate–perrhenate electrolyte with triethanolamine and monoethanolamine as surfactants.

Figure 11 shows that at low deposition current densities, the morphology of coatings obtained from baths with TEA and MEA additives differed insignificantly. Deposits from baths with MEA had a more densely packed structure. However, with an increase in deposition current density from 7.5 to 10 mA cm^−2^, all deposits, regardless of the type of ethanolamine added, acquired a distinctly globular structure (Figure 11g,h). The size of the globules, under otherwise identical conditions, was larger in the case of baths with TEA. Deposits from baths with MEA additives exhibited a textured structure. There is a significant difference in the morphology of deposits obtained at the same temperature (50 °C) from baths without (Figure 6b) and with ethanolamine additives (Figure 11). Ag-Re coatings deposited at a current density of 10 mA cm^−2^ from bath #5 with the same component composition but without additives (Figure 8b) were characterized by a finer-grained structure.

Following the outcomes of voltammetric studies, baths with a higher concentration of ethanolamine additives (bath #6 and #7) were chosen for electrodeposition.

#### 3.2.5. X-Ray Phase Analysis

To analyze the structure of coating obtained during the codeposition of silver with rhenium, Ag-Re samples with a higher (>1.5 wt.%) rhenium content were electrodeposited. Deposition was carried out from dicyanoargentate–perrhenate baths with a rhenium content up to ten times higher than silver (in a wider range of metal ion concentration ratios).

**Figure 12:**
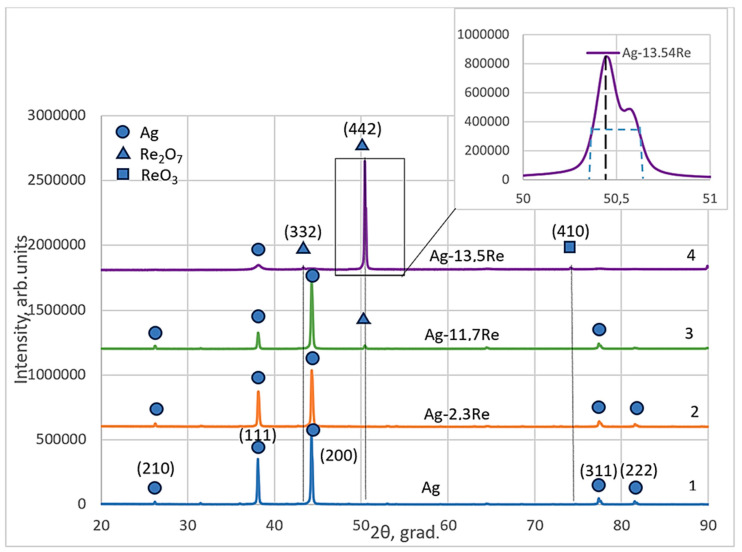
X-ray spectra electroplated silver (curve 1) and silver-rhenium coatings deposited from baths with different [Ag^+^]:[ReO_4_^−^] ratio: 10:1—2; 1:1.5—3; 1:10—4.

Structural analysis of phases and other structural parameters of electroplated silver-rhenium deposits and their comparisons with electrodeposited silver were performed using the XRD patterns that are shown in Figure 12. Diffraction patterns were obtained for coatings deposited at a current density of 30 mA cm^−2^ and a temperature of 18 °C from the baths #1, #2, #8, and #10, given in Table 1.

The obtained silver-rhenium coatings as well as silver are polycrystalline. The crystal structure of the electroplated Ag and Ag-Re alloys Ag-2.3Re, and Ag-11.7Re were investigated. The values of peaks at 38.12°, 44.28°, 77.47°, and 81.53° are attributed to silver crystal planes (210), (111), (200), (311), and (222) respectively (cf. Figure 12, curve 1–3). The Miller indexes in the diffraction pattern were assigned to each peak based on the data presented in JCPDS no 00–004-0783. The ratios of intensities (I_hkl_) of diffraction peaks depend on plating conditions and are shown in Table 3.

We previously determined [49] that silver deposits obtained from the BPC baths contain all possible textures like metallurgic silver exceptionally a texture (220). Two planes are predominant in electrodeposited silver, namely planes (111) and (200). Noticeably, the intensity of the peak revealed to the plane (200) is higher than to (111). This is typical for electrodeposited silver electroplated from the baths containing Ag(CN)_2_^−^. The intensity of other peaks corresponding to (311), (222), and (210) are negligibly low.

The obtained deposits have a strained lattice (not similar to metallurgic Ag [49]) with increased value of lattice parameter *a*_0_ from 4.077 Å for BPC electrolyte up to 4.096 Å for Ag-Re alloys regardless of the predominating of high packing planes (200) and (111) (Table 4). Probably, the differences in *a*_0_ values define the different properties of deposits.

For Ag-11.7Re and especially Ag-13.5Re coatings, the values of peaks at 43.48°, 50.46°, and 73.68° were indexed. These may be attributed to Re_2_O_7_ crystal planes (332) and (442), respectively (JCPDS no 04–007-0368). For Ag-13.5Re, the obtained peak at 73.68° corresponds to the ReO_3_ crystal plane (410) (JCPDS no 04–007-2352) (cf. Figure 12, curve 3, 4).

This is consistent with the results of the study [28], which established that increasing the concentration of perrhenate ions in the bath promotes the formation of rhenium oxides. However, considering the stepwise nature of the electroreduction process, the formation of an intermediate silver-rhenium(VI) oxide compound, such as Ag_x_ReO_3_, cannot be ruled out. Additionally, since the XRD analysis of our samples was conducted several weeks after electrolysis, the possibility that the diffraction peaks correspond to rhenium bronze-type compounds, H_x_ReO_3_, also cannot be excluded. In a study [50], structural transformations of ReO_3_ oxide and diffraction patterns for typical polycrystalline powder sample ReO_3_ recorded at different durations of time from the date of preparation were described. The additional peaks observed in such spectra (with 2theta values similar to ours) were attributed by the authors to the formation of H_x_ReO_3_ compounds over time. Thus, this issue requires further in-depth study.

**Figure 13:**
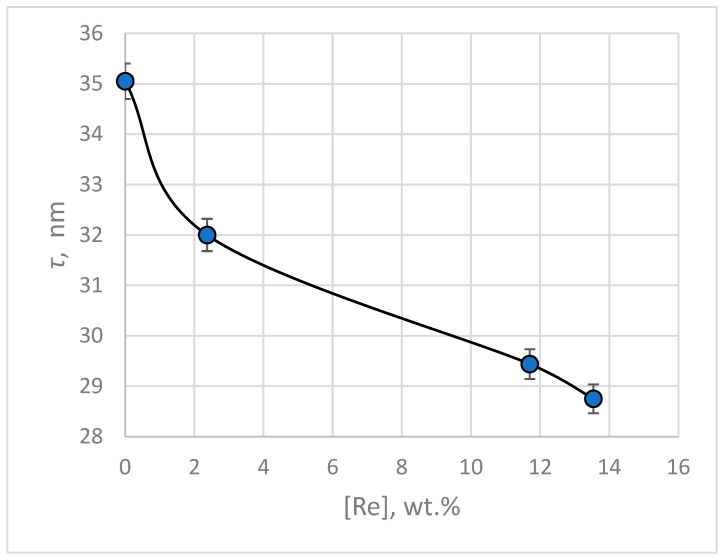
Average crystallite sizes of electrodeposited silver and silver-rhenium coatings depending on the rhenium content in the deposit.

The calculation of crystallite size was performed using the Scherrer Equation (3). The average values of crystallite size for electrodeposited silver and silver-rhenium coatings are presented in Figure 13. As follows from the presented data, the obtained electrodeposits are nanocrystalline.

The introduction of rhenium to silvering BPC electrolyte in an amount of 3–30 g L^−1^ leads to the grinding of the deposit and a decrease in the crystallites size from 35.05 to 28.75 nm at a current density of 3 mA cm^−2^. The refinement of crystallites is probably due to adsorbed particles that prevent the grain growth.

#### 3.2.6. Characterizing Coating Microhardness, Adhesion, and Porosity

**Figure 14:**
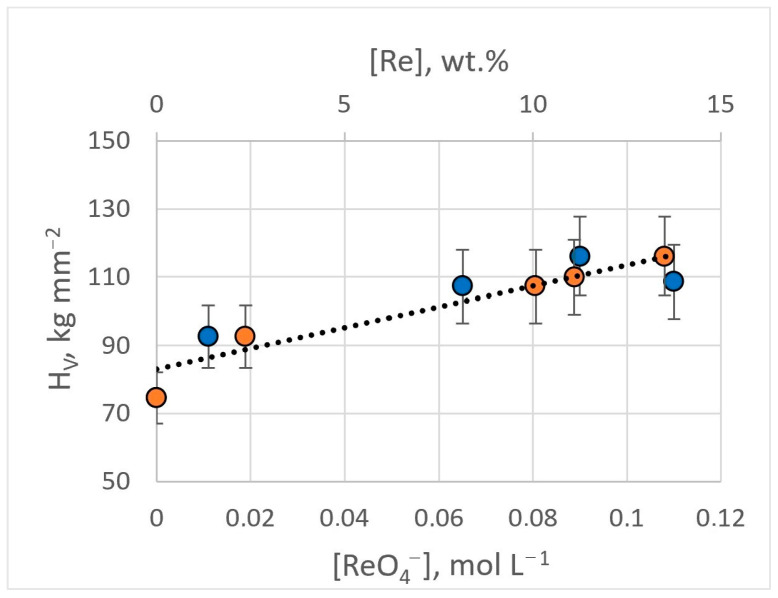
Dependence of the microhardness of Ag-Re coatings on the content of rhenium ions in the bath (blue dots) and the mass fraction of rhenium in the deposit (orange dots).

The microhardness of obtained Ag-Re coatings depends on the current density and the content of rhenium ions in the electrolyte. Silver coatings deposited from BPC electrolyte without rhenium ions in the current density range of 3–15 mA cm^−2^, have a hardness of 74.6 ± 9.7 kg mm^−2^. The introduction of 0.011 M KReO_4_ into the electrolyte (bath #2) leads to an increase in microhardness by 25% and higher, up to 92.6 ± 11.0 kg mm^−2^. Electrodeposition from dicyanoargentate–perrhenate baths with a higher [ReO_4_^−^]:[Ag^+^] ratio ensured an increase in microhardness. With [ReO_4_^−^]:[Ag^+^] ratio of 1.5:1 (bath #8), the microhardness value was 107.3 ± 16.0 kg mm^−2^. For the bath with [ReO_4_^−^]:[Ag^+^] ratio of 1.5:1 (bath #9, #10), coatings possessing a hardness of up to 116.1 ± 19.8 kg mm^−2^ were obtained (Figure 14). The H_V_ errors are not related to the measurement methodology but to the structural unevenness of the Ag-Re coating. The inclusion of rhenium in the coating provides greater stability of hardness over time.

**Figure 15:**
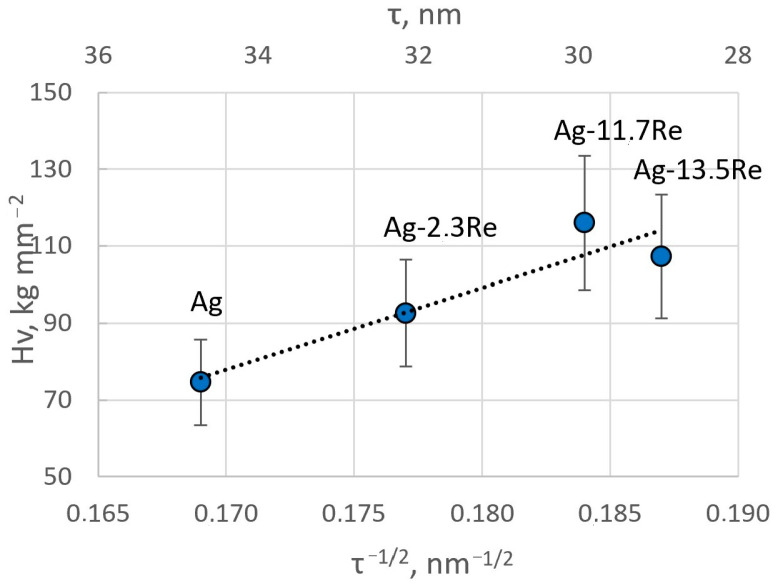
Hall–Petch plot for Ag-Re alloys showing the hardness as a function of the square root of the crystallite size determined from XRD measurements.

The obtained results are in good agreement with the crystallite size of the coatings determined from XRD data. The microhardness values are plotted in Figure 15 as a function of τ^−1/2^ (where τ is the crystallite size). A linear relationship, typical for Hall–Petch plots, is obtained, hence confirming that grain boundaries play a crucial role in the mechanical properties of these electrodeposited films. The decrease in crystallite size essentially creates a higher volume of grain boundaries and thus impedes the dislocation motion in these.

The adhesion strength of the deposits was determined using the marking method. A grid of 6 × 6 cuts was applied to the coating surface using a sharp blade. The cuts were made parallel and then at a 90° angle to form a grid. After cleaning the area with a brush, tape was applied and quickly removed. The adhesion was rated on a scale, where “0” indicates 100% adhesion and values from “1” (5% loss) to “5” (100% loss) represent decreasing adhesion.

The lattice cut results were similar across all samples, showing almost no visible signs of destruction or peeling at the cuts’ edges and intersections, indicating high adhesion to the substrates. Silver-rhenium coatings from all studied baths showed high adhesion to the copper substrate, scoring between “0” to “1” points, even at higher current densities. Silver coatings from rhenium-free electrolytes (bath #1) displayed slightly lower adhesion, scoring “2” to “3” points, suggesting that rhenium inclusion enhances adhesion.

Porosity (symbatic with corrosion resistance) was assessed using filter paper moistened with a solution (g L^−1^): K_3_[Fe(CN)_6_]—3, NaCl—10. Ag-Re coatings from bath #1 at current densities of 7.5–12.5 mA cm^−2^ and thicknesses of 6–8 µm were nearly non-porous. In bath #2, only at higher current densities were there an average of 3–5 pores per cm^2^. Bath #3 coatings showed porosity under similar conditions. Coatings from baths with ethanolamine additives were almost non-porous, potentially influenced by the substitution of rhenium salt with potassium perrhenate.

High corrosion resistance was noted for coatings from all baths. After a year in an air atmosphere, no color or property changes were observed, indicating their corrosion resistance. Furthermore, the absence of pores in the coatings corroborates their corrosion-resistant properties.

## 4. Conclusions

A new dicyanoargentate–perrhenate electrolyte has been developed for the electrodeposition of Ag-Re alloys, based on a borate–phosphate–carbonate buffering silver-plating bath. Silver-white Ag-Re alloys with a thickness of 2–15 µm, containing from 0.15 to 13.5 wt.% Re, were deposited from baths with [Ag^+^]:[ReO_4_^−^] ratios ranging from 10:1 to 1:10. Increasing the current density from 3 to 15 mA cm^−2^ tended to decrease the rhenium content in the deposit and reduce current efficiency from 98% to 66%, irrespective of the temperature (20–60 °C) and stirring. The coatings from Re-rich baths were more metallic and shiny.

Voltammetric studies were conducted in baths with varying [Ag^+^]:[ReO_4_^−^] ratios of 10:1, 2:1, and 1:5. The potential of the limiting current on the polarization curves for all studied baths was −0.65 V. Electrodeposition occurred under mixed kinetics, and the main kinetic parameters of the silver and rhenium co-deposition process were established based on the temperature factor. The calculated activation energy from exchange currents and inverse temperature was 65 kJ mol^−1^. The impact of ethanolamine additives (MEA and TEA) on the electrodeposition process was studied. TEA inhibited the electrode process, likely due to surface adsorption, while MEA activated the process and helped avoid silver anode passivation, possibly due to its complexing ability with silver (I) and the formation of a complex polyligand compound. The Re content in the alloy was lower in baths without surfactants.

X-ray analysis showed that the obtained Ag-Re coatings had a nanocrystalline structure with crystallite sizes ranging from 28.75 to 35.05 nm. The deposits obtained from Ag-rich baths had a strained lattice (dissimilar to metallurgic Ag) with an increased *a*_0_ from 4.077 Å for electrodeposited Ag up to 4.096 Å for Ag-Re alloys, regardless of the predominance of high packing planes (200). Additional peaks were recorded in the diffraction pattern for Ag-13.5Re coatings deposited from a Re-rich bath. These peaks may be attributed to ReO_3_ and Re_2_O_7_ crystal planes. To explain the peaks, taking into account the possibility of the formation of compounds such as Ag_x_ReO_3_ or H_x_ReO_3_, further study is required.

The microhardness of Ag-13.5Re deposits was more than one and a half times higher than that of pure silver (116.1 ± 19.8 vs. 74.6 ± 9.7 kg mm^−2^). Ag-Re coatings demonstrated high adhesion to the copper substrate and were practically non-porous. A correlation between the main structural parameters of the deposit and its properties (linear relationship “microhardness–crystallite size” in Hall–Petch coordinates) was revealed.

The “functional electrodeposition range” (j = 2.5–6 mA cm^−2^, t = 19–33 °C) of Ag-Re coatings with 0.7–1.5 wt.% Re as electrical contact materials operating in a temperature range from −196 °C to +250 °C has been established. Thus, the developed baths and electrolysis modes allow the electrochemical deposition method to synthesize functional Ag-Re coatings with a controlled, predetermined Re content, which are a promising material for use in the manufacture of electrical contacts operating in a wide temperature range.

## Figures and Tables

**Table 1:** The composition of dicyanoargentate–perrhenate baths used for the deposition of Ag-Re coatings.

Bath, (mol L^−1^)	#1	#2	#3	#4	#5	#6	#7	#8	#9	#10
NH_4_ReO_4_	-	0.011	0.022	0.0022	-	-	-	-	-	-
KReO_4_	-	-	-	-	0.055	0.055	0.055	0.065	0.09	0.11
KAg(CN)_2_	0.11	0.11	0.0044	0.022	0.11	0.11	0.11	0.044	0.009	0.011
KH_2_PO_4_	0.05	0.05	0.002	0.01	0.05	0.05	0.05	0.025	0.014	0.005
K_2_HPO_4_	0.3	0.3	0.01	0.06	0.3	0.3	0.3	0.15	0.085	0.03
K_2_CO_3_	0.025	0.025	0.001	0.005	0.025	0.025	0.025	0.001	0.0055	0.0025
H_3_BO_3_	0.2	0.2	0.007	0.04	0.2	0.2	0.2	0.1	0.05	0.02
C_2_H_7_NO (MEA)	-	-	-	-	-	0.033; 0.066; 0.099; 0.132; 0.160	-	-	-	-
C_6_H_15_NO_3_ (TEA)	-	-	-	-	-	-	0.015; 0.030; 0.045; 0.060	-	-	-
[Ag^+^]:[ReO_4_^−^] ratio	-	10:1	1:5	10:1	2:1	2:1	2:1	1:1.5	1:10	1:10
pH (at 20 °C)	7.00	7.65	7.80	7.60	7.21	8.78 (0.16 M MEA)	8.15 (0.06 M TEA)	8.18	8.42	8.49

**Table 2:** The stationary potential of a silver electrode in different compositions of electrolytes for the deposition of Ag-Re coatings (temperature 20 °C).

Bath	#1	#2	#3	#4	#5	#6	#7
E_Ag_, V (Ag/AgCl)	+0.200	−0.022	−0.122	+0.024	+0.007	+0.170	+0.110
E_Pt_, V (Ag/AgCl)	−0.0040	−0.0145	−0.030	+0.004	+0.004	+0.150	+0.0038

**Table 3:** Diffraction peak intensities in the XRD patterns for electrodeposited silver and silver-rhenium coatings.

Sample	Intensities I_hkl_ of Diffraction Peaks (in Arbitrary Units)			
	I_111_	I_200_	I_311_	I_222_
Ag	55	100	3	0.6
Ag-2.3Re	62	100	2	0.7
Ag-11.7Re	19.5	100	4	0.9
Ag-13.5Re	100	~0	~0	~0

**Table 4:** Lattice parameter (*a*_0_) and interplanar spacing (*d*_hkl_) for silver and silver-rhenium electroplated from various solutions.

		Bath #1	Bath #2 [Ag^+^]:[ReO_4_^−^] = 10:1	Bath #9 [Ag^+^]:[ReO_4_^−^] = 1:1.5	Bath #10 [Ag^+^]:[ReO_4_^−^] = 1:10
(111)	*Θ*, deg	19.07	19.06	19.06	-
	*d_hkl_*, Å	2.3567	2.3579	2.3579	-
	*a*_0_, Å	4.0770	4.0791	4.0791	-
(200)	*Θ*, deg	22.16	22.15	22.14	-
	*d_hkl_*, Å	2.0413	2.0422	2.0431	-
	*a*_0_, Å	4.0826	4.0844	4.0862	-
(311)	*Θ*, deg	38.72	38.74	38.74	-
	*d_hkl_*, Å	1.2309	1.2318	1.2318	-
	*a*_0_, Å	4.0824	4.0853	4.0853	-
(222)	*Θ*, deg	40.62	40.62	40.62	-
	*d_hkl_*, Å	1.1827	1.1827	1.1824	-
	*a*_0_, Å	4.0969	4.0969	4.0959	-
(442)	*Θ*, deg	-	-	-	25.22
	*d_hkl_*, Å	-	-	-	1.8071
	*a*_0_, Å	-	-	-	3.3202
(332)	*Θ*, deg	-	-	-	21.66
	*d_hkl_*, Å	-	-	-	2.0861
	*a*_0_, Å	-	-	-	2.2484

## Data Availability

The original contributions presented in this study are included in the article. Further inquiries can be directed to the corresponding author.
